# Dairy Products: Is There an Impact on Promotion of Prostate Cancer? A Review of the Literature

**DOI:** 10.3389/fnut.2019.00062

**Published:** 2019-05-14

**Authors:** Alexandra Vasconcelos, Teresa Santos, Paula Ravasco, Pedro Miguel Neves

**Affiliations:** ^1^Clinicas Viver, Lisbon, Portugal; ^2^European University of Lisbon, Lisbon, Portugal; ^3^Faculdade de Medicina, Instituto de Saúde Ambiental, Universidade de Lisboa, Lisbon, Portugal; ^4^Instituto de Ciências da Saúde, Universidade Católica Portuguesa, Lisbon, Portugal; ^5^University Hospital of Santa Maria, University of Lisbon, Lisbon, Portugal; ^6^Centre for Interdisciplinary Research in Health, Universidade Católica Portuguesa, Lisbon, Portugal

**Keywords:** prostate cancer, dairy products, milk, mTORC, Signaling of mTOR, IGF-1, BCAAs, cancer prevention

## Abstract

This review of the literature aims to study potential associations between high consumption of milk and/or dairy products and prostate cancer (PC). Literature is scarce, yet there is a direct relationship between mTORC1 activation and PC; several ingredients in milk/dairy products, when in high concentrations, increase signaling of the mTORC1 pathway. However, there are no studies showing an unequivocal relationship between milk products PC initiation and/or progression. Three different reviews were conducted with articles published in the last 5 years: (M1) PC and intake of dairy products, taking into account the possible mTORC1signaling mechanism; (M2) Intake of milk products and incidence/promotion of PC; (M3) mTORC1 activation signaling pathway, levels of IGF-1 and PC; (M4) mTORC pathway and dairy products. Of the 32 reviews identified, only 21 met the inclusion criteria and were analyzed. There is little scientific evidence that directly link the three factors: incidence/promotion of PC, intake of dairy products and PC, and PC and increased mTORC1 signaling. Persistent hyper-activation of mTORC1 is associated with PC promotion. The activity of exosomal mRNA in cellular communication may lead to different impacts of different types of milk and whether or not mammalian milks will have their own characteristics within each species. Based on this review of the literature, it is possible to establish a relationship between the consumption of milk products and the progression of PC; we also found a possible association with PC initiation, hence it is likely that the intake of dairy products should be reduced or minimized in mens' diet.

## Introduction

Prostate cancer (PC) is the most common in both sexes combined and the second most common cancer in males. This type of cancer will be diagnosed in one in each seven males (1). According to GLOBOCAN (2), in 2012 and all over the world, 1,1 million men diagnosed with PC which resulted in 307,000 deaths. This number corresponds to 15% of total cancers diagnosed in males and about 70% are registered in most developed regions. The PC incidence varies in all the world; however, the higher rates occur in Australia/New Zealand and North America (ASR 111,6 e 97,2 per 100,000). The incidence rate in western countries is very high and it can reach 80 to 100 cases per 100,000 persons per year. In Africa (North and East) and Eastern Asia, the incidence is lower: 10 to 20 cases per 100,000 persons.

In Portugal, according to the Portuguese Association of Urology, PC constitutes the second cause of death by cancer, right behind lung cancer, but is also the most frequent cancer in males above 50 years old. It is estimated an incidence of 82 cases per 100,000 inhabitants and a mortality rate of 33 per 100,000. PC represents about 3.5% of death causes and more than 10% of death by cancer.

PC incidence is higher in developed countries: There is a positive relationship between SIR (Standard Incidence Rate) and Development Index and its components, such as human life expectancy at birth, years of compulsory education and gross per capita income (3, 4). Another reason to justify this high PC incidence in developed countries may be connected with diagnosis improvement and the introduction of the routine evaluation of the specific antigen of PC (PSA) and the prostate biopsy, besides the population aging and the increase risk factors (5).

### Prostate Cancer and Milk/Diet

Western diet, rich in milk and dairy products, animal fat and sugars, with high calcium contents, is also connected to the risk growth of PC (6–8). Greater per capita milk consumption are probably correlated with higher PC incidence and mortality according World Cancer Research Foundation 2007 (9–13).

Milk consumption has always been associated to child mortality reduction, increased fertility, increase of children's BMI and of adolescence growth (14–16). Milk, relevant sources of proteins and other macromolecules provides various nutrients and bioactive molecules to support their growth and development (15). However, milk consumption only in adolescence was associated to an increase of 3.2 times the risk of advanced PC in the cohort population of 8894 Icelandic men (13).

According to the conclusions of a 2016 meta-analysis pursued by investigators from the Republic of China, the ingestion of dairy products has no significant impact in the mortality risk increase in all kind of cancers while a small daily ingestion of dairy products can even reduce the risk based on a non-linear module (17). However, the same study concludes fat milk consumption by men may contribute significantly to the increase of PC mortality risk. There is thus a direct relationship between milk ingestion and increased PC mortality (17, 18).

According to some authors, the increase of 35 gr./per day of milk proteins consumption was associated to a 32% increase of PC risk (19). In some Cohort studies, results are less conclusive regarding milk negative impact (20–22). According to Nacional Cancer Institute (USA), more than 2.6 million people in USA are PC survivors and they have potentially improved the prognosis by adopting healthy lifestyle habits (23). Limit the consumption of meat and dairy products, particularly high fat, is part of the recommendations to reduce the risk of PC progression (24).

### Importance of Androgen Receptor for Prostate Cancer

Androgen receptor (AR) mediated signaling is required for the proliferation of prostate cancer cells and is a critical receptor for the development and progression of prostate (25). Prostate cancer is dependent on androgen receptor signaling (25). The high concentration of steroid hormones present in dairy products may, very probably, be related to the effect these products have on the initiation and promotion of prostate and breast cancer. (26). Dairy products concentrate hormones as insulin-like growth factor-1 (*IGF-1*). (27–29). In recent years, according to epidemiological evidence, the risk of colon, pancreatic, endometrial, breast, and prostate tumors is associated with high IGF-1 levels (30) and increased IGF/IGF-1R signaling is implicated in all stages of carcinoma progression (31, 32). On the other hand, according to a recently published review article, the scientific evidence points to the intake of milk and dairy products that meets the recommended nutrients that protect against the most prevalent chronic diseases, however, the evidence for PC is inconsistent (33). The androgen receptors and the signaling mechanism of rapamycin complex 1 (mTORC1) and PI3 kinase-AKT may be key points in the PC. (34, 35) and its dysregulation is common in many human cancers such as prostate, colon, breast and thyroid (36). The objective of this review of the literature is to evaluate a potential correlation between milk and dairy consumption and the incidence and progression of prostate cancer, given that:

- The signaling pathway of mTORC1 and P13 Kinase-AKT is a cancer-promoting mechanism (33, 37–42).
- Signaling of mTOR complex1 can be activated by BCAAs (leucine), Glutamine, Insulin, IGF-1, glucose, ATP (43–47).
- Milk provides nutrients that can activate mTORC signaling pathways (48, 49).

## Materials and Methods

### Information Sources and Keywords

A search was performed in the PubMed (US National Library of Medicine) using the terms MeSH (Medical Subject Headings) identified and corresponding to the following keywords: prostate cancer, dairy products, milk, IGF, mTORC1.

The various searches were performed using only MeSH terms:

“Prostatic Neoplasms”[MeSH], “Dairy Products”[MeSH], “Cultured Milk Products”[MeSH], “mTOR protein, human” [Supplementary Concept], “Insulin-Like Growth Factor I”[MeSH], “mechanistic target of rapamycin complex 1” [Supplementary Concept], “TOR Serine-Threonine Kinases”[MeSH].

**Figure 1:**
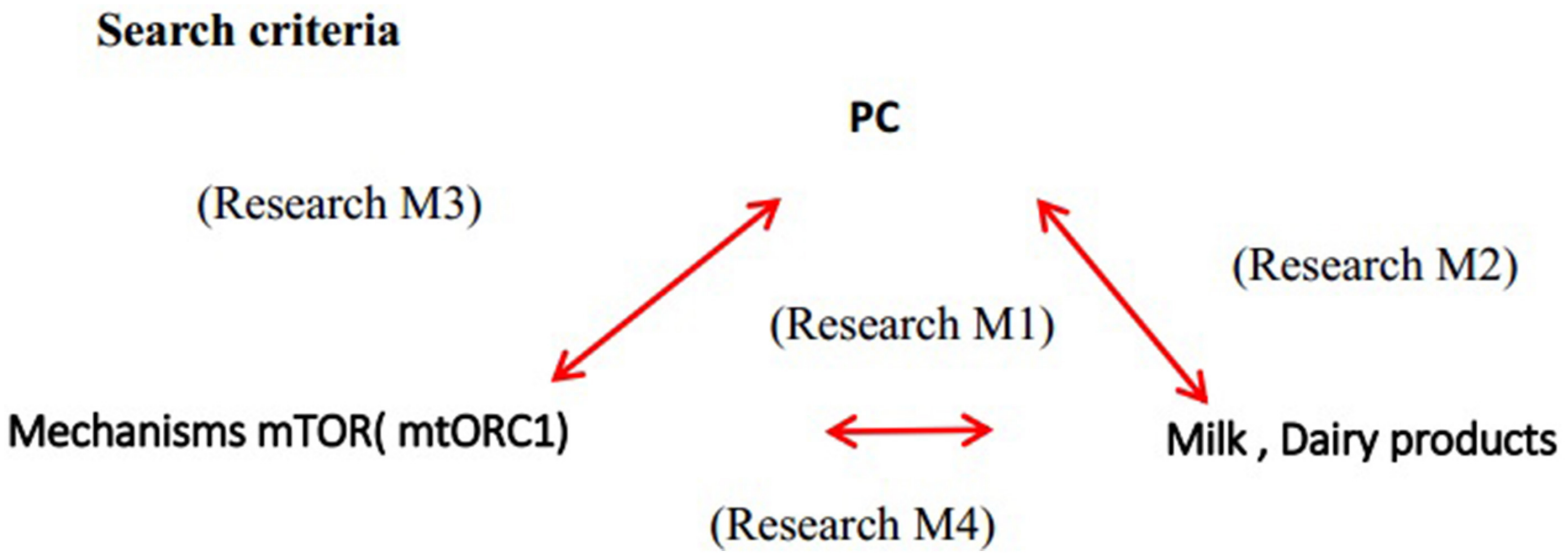
Relationship between various searches.

The research was done according to the following research criteria relating the factors as follows (Figure 1):

- Relate the incidence of PC to the consumption of dairy products taking into account the possible signaling mechanism of mTORC1. **Research M1**- with “Prostatic Neoplasms” (MeSH) AND (“Dairy Products” [MeSH] OR “Cultured Milk Products” [MeSH]) AND (“MTOR protein, human” [Supplementary Concept] OR “Multiprotein Complexes”] OR “Insulin-Like Growth Factor I” [MeSH])
- Relate the intake of dairy products with the incidence of PC-**Research M2**—with “Prostatic Neoplasms” (MeSH) AND (“Dairy Products”) OR “Cultured Milk Products” (MeSH).
- Relate the possible mechanism of activation of the mTORC1 signaling pathway, IGF1 levels with the PC-**Research M3** with (Insulin-Like Growth Factor I [MeSH] OR mtor protein [MeSH Terms] OR mechanistic target of rapamycin complex 1 [Supplementary Concept] OR “TOR Serine-Threonine Kinases” [MeSH]) AND (prostate cancer [MeSH Terms] OR “Prostatic Neoplasms” [MeSH]).
- Relate the mTORC mechanism to dairy products—**Research M4** with dairy products [MeSH Terms] OR “Cultured Milk Products” [MeSH]) AND [“mtor protein [MeSH Terms]” OR mechanistic target of rapamycin complex 1 [MeSH Terms]) OR “TOR Serine-Threonine Kinases” [MeSH]).

### Eligibility and Exclusion Criteria

The research was elaborated according to the following eligibility criteria: Inclusion of review articles and systematic reviews, published in the last 5 years, that restricted research to the human species, male adults, as a target population and also delimiting the research Cancer awareness. All articles were manually analyzed and eligible studies should show association between consumption of dairy products, PC and mTORC1 and/or IGF. Those whose content, despite including MeSH terms defined, deviated from the objective of this review of the literature, because they were out of the subject or because they were repeated. Eligible articles are included in this review of the literature.

## Results

As a result of the various surveys, 32 articles were reviewed and systematic reviews divided by the groups (Table 1). **Research M1-** No review or systematic review with the research was identified in the database using the MeSH terms that relate the consumption of dairy products to PC and to the mechanism of activation of the mTORC1 signaling pathway. Although the search with MeSH terms does not present any publication, it was found that doing a **search (Kw)** using the keywords (mTORC1) and (prostate cancer) and (milk), and not the MeSH terms, there is a review within the scope of the Work that relates cow's milk to mTOR signaling and promotion and initiation of PC.

**Table 1:** Search Results (Kw–search with keywords and no MeSH terms).

	**Search criteria**	**Number of articles excluded**	**Excluded references**	**Number of articles included**	**Included references**
**Research M1**	PC to the consumption of dairy products taking into account the possible signaling mechanism of mTORC1.	0		0	
**Research M2**	Relate the intake of dairy products with the incidence of PC.	4	(50–53)	5	(54–58)
**Research M3**	Relate the possible mechanism of activation of the mTORC1 signaling pathway, with the PC.	6	(59–64)	10	(65–74)
**Research M4**	Relate the mTORC mechanism to dairy products.	1	(75)	6	(76–81)
**TOTAL**		**11**		**21**	
**Research Kw**	PC to the consumption of dairy products taking into account the possible signaling mechanism of mTORC1.	0		1	(82)

**Research M2**−9 articles were identified that relate the consumption of dairy products to PC, of which 4 were excluded because they were included in the exclusion criteria. Of these 4 studies, 3 were excluded because they related dairy consumption to other specific cancers and not to PC or cancer in general (50–53).

**Research M3**—With the research, 16 reviews and systematic reviews were identified that relate PC to mTORC1 and IGF mechanisms. These subjects were excluded from the study by 6: 3 because they addressed the issue of PC radiation (59–64).

**Research M4**—With the research, 7 systematic reviews and reviews were identified. In this research the filters “human species, man and cancer” were not used because they did not apply to the subject in question. We excluded 1 review article general (75) for being out of topic.

## Discussion

### Dairy Products and Prostate Cancer (Research M2)

Of the 5 eligible studies, 1 shows no relationship between dairy intake and cancer. It also concludes that dairy intake reduces the risk of colorectal cancer and is silent about PC (54). The remaining 2 studies associate Dairy Products consumption with increased risk and PC incidence (55, 56). 2 studies conclude that PC prevention strategies go through moderation in consumption of food, meat and dairy products (57, 58).

The first publication analyzed (54) concludes that there was no consistent association between the consumption of dairy products and all specific or non-specific causes of mortality. In this systematic review were identified 408 studies of which excluded 384 by the analysis of the title and abstract not to be included in the theme of the review and excluded another 12 studies after analysis of its content. Of the 13 remnants the majority did not show consistent evidence on the relationship between mortality and milk consumption (54).

An *in-vivo* study with 2 laboratory guinea pigs showed that the consumption of high doses of milk did not produce PC progression in early stages of tumorigenesis. In addition, the authors conclude that regular milk consumption should not be considered detrimental for patients with early prostatic tumors (83). However, another *in vitro* study shows that milk can stimulate the growth of prostate cancer cells in culture (84).

Another review and meta-analysis (56) concluded that ingestion of high amounts of dairy products, milk, low-fat milk, cheese, yogurt, and calcium from dairy rather than from supplements or sources Non-dairy products, may increase the overall risk of prostate cancer. This review identified 45 relevant studies, excluding 13 studies per repetition, comparing only 3 types of dairy products, when the objective would be all types of dairy, or for moving away from the theme. The authors also concluded that the divergent results for different types of dairy products and calcium sources studied suggest that other components in dairy products, in addition to fat and calcium, may be related to the increased risk of PC (56).

The high intake of dairy products was also associated with PC with some consistency. However, the same review (55) reports a meta-analysis with 4 cohort studies that shows that there is no evidence between intake of calcium added to dairy products and PC (85). In their conclusions, they are vague in this regard, warning of the need for further studies to clarify the mechanisms involved in carcinogenesis linked to these products (55, 85).

Intake of calcium through consumption of dairy products and not through calcium provided by non-dairy products, showed a positive association with the overall risk of prostate cancer in the studies (55, 84–88). However, there was a divergence in results that may be related to other components in dairy products (65). Furthermore, the hypothesis that calcium from dairy products may increase PC risk, does not explain the non-increased risk with ingestion of non-dairy calcium sources (19, 87). Calcium intake is positively associated with aggressive PC while vitamin D intake shows an inverse relationship, and this association varies according to race and BMI (89).

In this review of the literature, 2 studies were also analyzed that are related to PC prevention strategies and both conclude that there is some evidence that moderate food consumption, reduction of dairy intake, Asian or Mediterranean diet may help prevent PC associated with other factors such as healthy habits (57, 58). There is very significant evidence supporting the association between the Mediterranean diet and the reduction of PC risk and disease progression (90, 91).

### mTOR, mTORC1, IGF-1, and Prostate Cancer (Research M3)

Six studies conclude that deregulation of mTOR pathway signaling is present in most PC and shows evidence for possible therapy in castration-resistant or hormone-resistant PC with inhibitors of these pathways (66–71); 3 studies relate circulating amounts of IGF with PC progression (72–74); 1 Study associates positively the metabolic syndrome/IGF and PC incidence (65).

Deregulation of the mTOR signaling pathway is associated with the promotion or progression of PC and evidence for possible PC therapy resistant to castration or hormone-resistant with inhibitors of these pathways(69, 71). Rapamycin was the first mTOR inhibitor discovered and approved for the treatment of cancer. Thus, inhibitors of these pathways may be therapeutic alternatives for PC; However, most are unstable molecules that exhibit toxic effects, and further studies are needed (67, 68, 71). On the other hand, fisetin, a dietary flavonoid, may act as a double inhibitor of the PI3K/AKt and mTORC pathways. It is a significant finding that the activation or overexpression of mTOR signaling is common in tumors with overexpression of Pi3K/Akt (66).

MTOR inhibitors may be a promising therapy in PC that is resistant to conventional hormone therapy (67). The mTOR protein plays a crucial role in this signaling pathway being responsible for the regulation of cell growth and protein synthesis. Changes in this pathway are associated with carcinogenesis, angiogenesis, tumor growth and metastasis, and this deregulation is present in almost 100% of the advanced PCs (69, 92). Preclinical studies demonstrate that the PI3K/Akt/mtORC signaling pathway plays a key role in the progression of castration-resistant PC (68, 70). Recent literature shows that mTORC1 controls the programming in mRNA translation of the genes involved in the initiation and metastasis of PC (93–95). Reviews also relate PC to circulating IGF amounts. IGF, essential for physiological growth, may also be implicated in numerous diseases, including cancers. Anabolic signs of IGF-1 may promote the development of tumors through the anti-apoptosis effect and by stimulation of cell proliferation (96, 97). Modulators of this factor, such as IGFBP-6, may be useful in modeling the amount of IGFII (73). Preclinical studies in PC show that the segmentation of IGF receptors may constitute a promising anti-tumor effect (74). The association between obesity/insulin resistance and PC progression and lethality may be mediated through the activation of the GH/IGF1 axis (72). Therapies that inhibit this axis may play a preventive role in the progression of the disease (74). There is a direct correlation between the metabolic syndrome, hyperinsulinemia and elevated levels of IGF and PC(65). A recent meta-analysis of 12 studies found a slightly increased risk for PC incidence (odds ratio 1.38, 95% CI 1.19–1.60) in subjects with higher serum IGF-1 levels. The authors have some limitations, since they only use a single blood collection per patient, the titration was performed only by laboratory methods, and in some patients the stage of disease progression was not known (98). However, epidemiological studies also show that higher levels of circulating IGF-1 within normal ranges are associated with increased risk of developing tumors including PC, colorectal and breast cancer (99). If we analyze the influence of increased mTOR pathway signaling on PC promotion, there is a great deal of clear information and even many revisions that show that the path of therapy may be related to this mechanism. In almost 100% of PC there is deregulation of this signaling pathway (92).

### Dairy Products and Increased TOR, PI3 Kinase-AKT Signaling (Research M4)

The 6 eligible publications reflect a positive association between milk consumption and increased mTORC1 signaling. Human milk contains 1.2 g/100ml of milk proteins compared to cow's milk containing 3.5 g/100ml, almost 3 times more branched-chain amino acid proteins (BCAAs) (77, 79, 100–102), especially leucine (103). The available BCAAs play a key role in the activation of the mTORC1 complex (104–106). Glutamine in milk, 70% more than in meat (107) is also an amino acid with important activation power of mTORC1 (108, 109). In addition, pasteurized cow's milk transfers biologically active exosomal microRNA to the systemic circulation of the consumer that apparently can affect more than 11,000 human genes, including the mTORC1 complex (80). Breast milk has an essential role in regulating growth during the postnatal period of mammals (110) and constitutes a complex bio-fluid that transfers nutritionally important, protective and fundamental substances for the optimal developme nt of the baby (62). However, their intake during adolescence seems to be being called into question. Excess nutrients from dairy products ingested systematically since adolescence, such as glutamine, BCAAs, leucine, palmitic acid, IGF, bioactive exosomal microRNAs promote an over-activation of the mTORC1 complex with consequent increase in transcription, translocation of mRNA, cell growth and proliferation cellular (44, 105, 106, 111–113).

Milk may represent the most sophisticated endocrine regulation system for the activation of mTORC1 complex (78) for the supply of BCAAs from milk proteins and exosomal mRNA produced by mammary glands that contribute to the activation of mTORC1 related to Numerous modern diseases (114, 115). Excessively ingested milk may be an epigenetic amplifier of the FTO gene-mediated transcription (79). The FTO gene (association and obesity and fat mass) is assigned a fundamental role in the control of body weight, body composition and energy balance (116). Milk, the most common food introduced from an early age, is not only a food but also represents a sophisticated signaling system that promotes mTORC-mediated growth (78) and has also been shown to stimulate mTORc1-dependent translocation (80). The increased expression of the milk-activated FTO gene leads to mTOR deregulation and increased mRNA translation (117). Individuals with this epigenetic alteration may be more susceptible to milk-mediated FTO activation (79). In addition, there is also a positive correlation between circulating IGF-1 levels and milk consumption. After the meta-analysis, the weighted mean difference in circulating IGF-I level was 13.8 ng/ml (95% confidence interval: 6.1–21.5 ng/ml) comparing the intervention group with the control group (32). However, factors such as insulin, IGF and isolated amino acids do not appear to be sufficient for maximal activation of TORC1 (118). On the other hand, insulin in the presence of amino acids from dairy products can induce a maximal activation of mTORC1 (119). Other reviews analyzed, relate the consumption of dairy products and the activation of the mTORC signaling pathway and conclude that milk consumption during pregnancy can be determinant in the risk of developing diseases of civilization, such as obesity, diabetes, and cancer (76) and therefore the limits of milk consumption during this phase should be reevaluated (120).

The exaggerated increase in western dietary mTORC1 signaling may explain the association between diabetes mellitus (81) and acne (121) with increased fat mass, insulin resistance (122) and early menarche (15). They may also be indicators of over-activation of the mammalian target of rapamycin 1 complex, arterial hypertension (38, 123–125).

### Dairy, Prostate Cancer, and Increased mTOR Signaling (Research KW)

No review was found with the selection criteria that directly related the incidence of PC with increased signaling of the mTORC pathway induced by dairy products. However, the identified study where the keywords were used and not the MeSH terms (Kw research), relates the impact of cow's milk on mTORC1 signaling on PC initiation and progression (82). PC is dependent on androgen receptors and aberrations in PI3K/Akt-mTORC1 pathways, by excessive signaling. This activation enhances mRNA transcription and distinct phases of PC initiation and progression. Some components of cow's milk, not breast milk, can activate mTORC. Increased intakes of cow's milk, dairy proteins, and estrogens in cow's milk, especially of pregnant cows, may explain the high incidence of PC in modern Western societies. Milk proteins contribute to the increase of branched-chain amino acids (BCAAs—leucine, isoleucine, and valine) and elevated postprandial plasma levels of IGF-1 insulin, which activate mTORC-signaling pathways (82, 126, 127). Higher levels of glucose and ATP may also be implicated in this deregulation (44, 128). However, studies show evidence that only whole milk may be associated with a high incidence of lethal PC (129). The FTO gene is associated with increased PC risk (130, 131). Milk-mediated epigenetic activation of FTO can lead to FTO activation leading to the activation of mTORC1 (65) and possibly related to the onset and metastasis of PC (82, 95). Consumption of dairy products may increase this signaling, since current milk is concentrated in the nutrients that contribute to its over-activation. The activation of mTORC1 depends on available amounts of AAs, specifically BCAAs and leucine, glutamine, IGF, insulin, exosomal micro mRNA, palmitic acid, high glucose levels, and ATP (44, 80, 128). However, it should be clarified which type of dairy and what concentrations in hormones, leucine, IgF, BCAAs required for mTORC activation and synergistic combinations of potentiating nutrients (119). The origin of milk (cow, goat, sheep, and pregnant cows), type of dairy products and amounts of intake may imply different impacts on the increased risk and progression of PC (132). The studies analyzed do not differentiate this question. According to the studies, the impact on risk may also be dependent on the time at which dairy consumption is higher, and in the postnatal period, the benefits of breast milk consumption are unequivocal, whereas during adolescence the consumption of milk from cow may contribute to the increased risk of PC (13). Exosomal mRNA activity in cell communication may lead to different impacts of different types of milk and whether or not mammalian milks will have their own characteristics within each species. Can the microRNAs contained in cow's milk induce this change in other mammals?

Some polymorphisms may also be decisive in PC expression, such as FTO (65, 79). Is this increase in signaling dependent on some other polymorphism?

Eligible studies in this research have some limitations. The definition of search criteria is very extended, there was substantial heterogeneity among studies of non-fermented milk consumption in relation to mortality from all causes. Nor have any reviews considered the different types of dairy products, diverging results for types of dairy products and sources of calcium suggest that other components of dairy than fat and calcium may increase prostate cancer risk.

Some authors conclude that moderate food consumption, reduction of dairy products can prevent prostate cancer but also harbors additional beneficial effects on general health. However, moderate consumption and the kind of dairy Products are not defined. No review correlates with other concomitant eating habits, genetic factors, or lifestyle.

## Conclusions

Studies reviews selected through defined research criteria that directly relate to dairy consumption and PC are contradictory, inconsistent, and omitted in possible justifications or responsible mechanisms. Nevertheless, most of the reviews point to a positive association between milk consumption and the incidence or increase of PC risk. However, there is strong evidence in the literature (not reviews) that associates dairy consumption and PC (91) and disease progression (82, 133), and possibly initialization (82). Through this review of the literature we understand the different mechanisms triggered by nutrients contained in milk and derivatives that may lead to an increased risk of prostate cancer in its consumers. However, further studies are needed to clarify which detrimental derivatives and which quantities are required for tumor progression and/or initiation. A better understanding of cancer and this signaling pathway may lead to the development of more effective and tumor-specific drugs and to define preventive measures of the tumor with a higher incidence in man and other types of cancer, such as breast cancer (67).

In conclusion, according to the analysis of the available information, it is possible to establish a relationship between the consumption of dairy products and the progression of PC and possibly its initiation and should therefore be reduced or minimized in the diet of men.

## Author Contributions

AV conceived the study, participated in its design and coordination, draft, and authored the manuscript. PR participated in the study design, interpretation of the data, and helped to draft manuscript revisions. TS and PN were responsible for scientific writing and manuscript editing.

### Conflict of Interest Statement

The authors declare that the research was conducted in the absence of any commercial or financial relationships that could be construed as a potential conflict of interest.

## Abbreviations

PC: Prostate Cancer
mTORC1: Mammalian Target of Rapamycin Complex
P13K: phosphatidylinositol 3-kinase
Akt: Proteins kinase
IGF 1: Insulin-like Growth Factor
BCAAs: Branched chain amino acids
T2DM: Type 2 Diabetes Mellitus
Aas: Amino acids
BMI: Body Mass Index.
